# The Curcumin Analog PAC Is a Potential Solution for the Treatment of Triple-Negative Breast Cancer by Modulating the Gene Expression of DNA Repair Pathways

**DOI:** 10.3390/ijms24119649

**Published:** 2023-06-02

**Authors:** Esraa Almalki, Abdullah Al-Amri, Reem Alrashed, Mohamed AL-Zharani, Abdelhabib Semlali

**Affiliations:** 1 Department of Biochemistry, College of Sciences, King Saud University, Riyadh 11451, Saudi Arabia; almalkiesra@gmail.com (E.A.); abdullah@ksu.edu.sa (A.A.-A.); alrashedre@gmail.com (R.A.); 2 Biology Department, College of Science, Imam Mohammad Ibn Saud Islamic University (IMSIU), Riyadh 11623, Saudi Arabia; mmyalzahrani@imamu.edu.sa; 3 Groupe de Recherche en Écologie Buccale, Faculté de Médecine Dentaire, Université Laval, Québec, QC G1V 0A6, Canada

**Keywords:** breast cancer, DNA repair pathway, curcumin analog, PAC

## Abstract

Breast Cancer (BC) is one of the most common and challenging cancers among females worldwide. Conventional treatments for oral cancer rely on the use of radiology and surgery accompanied by chemotherapy. Chemotherapy presents many side effects, and the cells often develop resistance to this chemotherapy. It will be urgent to adopt alternative or complementary treatment strategies that are new and more effective without these negative effects to improve the well-being of patients. A substantial number of epidemiological and experimental studies reported that many compounds are derived from natural products such as curcumin and their analogs, which have a great deal of beneficial anti-BC activity by inducing apoptosis, inhibiting cell proliferation, migration, and metastasis, modulating cancer-related pathways, and sensitizing cells to radiotherapy and chemotherapy. In the present study, we investigated the effect of the curcumin-analog PAC on DNA repair pathways in MCF-7 and MDA-MB-231 human breast-cancer cell lines. These pathways are crucial for genome maintenance and cancer prevention. MCF-7 and MDA-MB-231 cells were exposed to PAC at 10 µM. MTT and LDH assays were conducted to evaluate the effects of PAC on cell proliferation and cytotoxicity. Apoptosis was assessed in breast cancer cell lines using flow cytometry with annexin/Pi assay. The expression of proapoptotic and antiapoptotic genes was determined by RT-PCR to see if PAC is active in programming cell death. Additionally, DNA repair signaling pathways were analyzed by PCR arrays focusing on genes being related and confirmed by quantitative PCR. PAC significantly inhibited breast-cancer cell proliferation in a time-dependent manner, more on MDA-MB-231 triple-negative breast cancer cells. The flow cytometry results showed an increase in apoptotic activity. These data have been established by the gene expression and indicate that PAC-induced apoptosis by an increased Bax and decreased Bcl-2 expression. Moreover, PAC affected multiple genes involved in the DNA repair pathways occurring in both cell lines (MCF-7 and MDA-MB231). In addition, our results suggest that PAC upregulated more than twice 16 genes (ERCC1, ERCC2, PNKP, POLL, MPG, NEIL2, NTHL1, SMUG1, RAD51D, RAD54L, RFC1, TOP3A, XRCC3, XRCC6BP1, FEN1, and TREX1) in MDA-MB-231, 6 genes (ERCC1, LIG1, PNKP, UNG, MPG, and RAD54L) in MCF-7, and 4 genes (ERCC1, PNKP, MPG, and RAD54L) in the two cell lines. In silico analysis of gene–gene interaction shows that there are common genes between MCF-7 and MDA-MB-321 having direct and indirect effects, among them via coexpression, genetic interactions, pathways, predicted and physical interactions, and shared protein domains with predicted associated genes indicating they are more likely to be functionally related. Our data show that PAC increases involvement of multiple genes in a DNA repair pathway, this certainly can open a new perspective in breast-cancer treatment.

## 1. Introduction

Breast cancer (BC) is a major global public health problem being multifactorial and caused by genetic alteration and environmental factors that lead to 2.2 million incident cases and approximately 685 thousand deaths in women worldwide, in 2020. It is now a fatal disease and is considered the second most frequently diagnosed cancer [1]. The conventional treatments consist of using hormonotherapy, chemotherapy, and radiotherapy. Although these seem relatively effective, they have many side effects that make life very difficult for patients having this type of disease, as multidrug resistance [2,3] and unselective therapies are the main obstacles in the treatment of breast cancer, which harshly affect normal cells. Consequently, it is needful on an urgent basis to face this deadly situation by developing an alternative or a complementary safe and effective treatment system for breast cancer. Historically, the discovery of medicinal plants has had a significant influence on human health; 50% of all new medications approved from 1981–2014 were directly related to natural products. In the anticancer area, only 17% of the total drugs were purely synthetic while 83% were either natural products or designed-based [4]. Curcumin is a promising agent to treat cancer as well as other diseases. It can modulate a wide range of targets involving transcription factors (e.g., NF-κB, STAT3, AP-1), kinases (e.g., EGFR, ERK, JAK, and APK), and cytokines (e.g., TNF, ILs, MIP, and MCP) [5]. Due to its anticancer effect and safety property, curcumin and its analogs could serve as potential effective agents in many types of therapies [6]. Due to practical constraints, the limited distribution to the target tissues, and the low plasma levels of curcumin, alternative strategies have been explored to overcome its pharmacokinetic limitations. Even though curcumin had anticancer effects, it failed clinically. As a result, scientists have a better strategy for a fatal illness such as cancer; moreover, to circumvent its limitations and increase its efficiency, chemical modifications have been done to its structure leading to a wide range of analogs being synthesized [5,7]. Many studies summarized the information and tested the activity of several curcumin analogs against different illnesses. A lot of these had anticarcinogenic potential [8]. One of them (i.e., PAC) was investigated in this existing study. A novel curcumin analog made by Prof. Youssef, 3,5-bis (4-hydroxy-3-methoxy benzylidene)-N-methyl-4-piperidone (PAC), has recently been studied to improve its chemopreventive efficacy and have better cancer therapeutics by inducing its low systemic bioavailability [9]. In an animal model, it has been shown that PAC had improved bioavailability and greater stability in the blood compared to curcumin [10,11,12]. These studies provide valuable insights into the development of PAC where its role in cancer treatment and prevention needs to be explored.

In response to DNA damage, a series of signaling cascades is activated to promote cell survival. The impact of DNA repair pathways on cancer biology and therapy was documented and considered a key regulator of tumorigenesis [13]. Systems for DNA repair are triggered to maintain the genetic stability of cells and their integrity following exposure to various endogenous/exogenous DNA damage sources by minimizing the consequence of detrimental mutations [14]. DNA repair is an important line of defense against mutation. In this way, DNA repair-system defects can lead not only to the initiation and progression of cancer but may also contribute to hypersensitivity or resistance of cancer cells to genotoxic agents [15]. Targeting the DNA repair pathway may be a potential therapeutic approach for cancer treatment by increasing the tumor sensitivity to drugs. Although different studies demonstrated the anticarcinogenic effect of PAC, its underlying role in DNA repair pathways was not elucidated. Consequently, in the present study, we explore its contribution by investigating these pathways in human breast-cancer cell lines MCF-7 and MDA-MB-231.

## 2. Results

### 2.1. PAC Induces Morphological Changes in MCF-7 and MDA-MB-231 Cells

**Figure 1:**
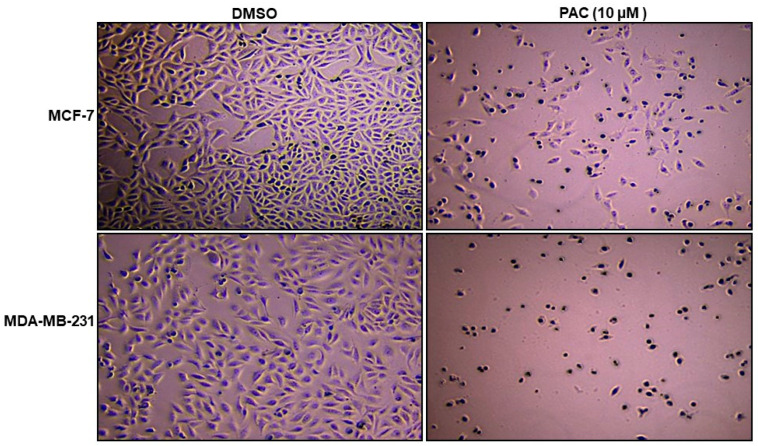
Effect of PAC on cell morphology in MCF-7 and MDA-MB 231 cells. Cells were treated with DMSO and PAC at a concentration of 10 μM for 24 h. The results were observed under an inverted microscope; magnification, ×20.

Differences in cell morphology were observed between the PAC-treated cells and the controls (with DMSO treatment) by light microscopy. As demonstrated in Figure 1, the most dramatic changes in morphology were seen in PAC-treated cells, manifested by cell shrinkage, becoming round, and loss of their cell integrity, which promotes the cancer-cell progression as well as a significant reduction in cell numbers and their extensive detachment from the substratum in culture compared with the vehicle.

Morphological changes were more visible in MDA-MB-321 than MCF-7, probably characteristic of breast-cancer cell death and apoptosis. They were more remarkable when the cells were under long-term treatment with PAC or with concentrations > 10 μM.

### 2.2. PAC Inhibits Cell Proliferation and Potent Cytotoxicity against MDA-MB-231 and MCF-7 Cells

**Figure 2:**
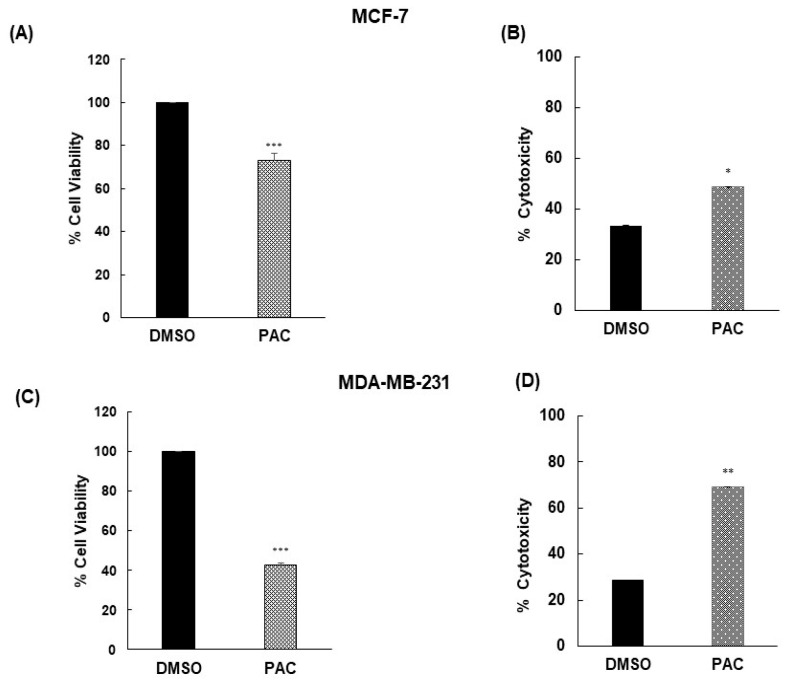
Effect of PAC on MCF-7 and MDA-MB 231 cell viability and cell toxicity. (**A**,**C**) Cell viability was determined by the MTT assay respectively in MCF-7 and MDA-MB-231 cells. Cell viability was calculated as a ratio (percentage) of the number of viable cells in the experimental wells to that in the control well. Cells were initially seeded at the density of 3 × 10^3^ per well, the results are expressed as mean ± S.D. (n = 8). *, *p* < 0.05; **, *p* < 0.01; ***, *p* < 0.001 versus control. (**B**,**D**) Cell cytotoxicity respectively in MCF-7 and MDA-MB 231 cells was used by LDH assay. Cells were treated with DMSO or PAC at a concentration of 10 μM for 24 h. The results are expressed as mean ± S.D. (n = 3). *, *p* < 0.05; **, *p* < 0.01 versus control.

The antiproliferative activity of PAC was evaluated by MTT assay after 24 h in the treatment of MCF-7 and MDA-MB-231. We observed that in both, there was a clear trend of decreasing cell viability in those treated while an increase in proliferation was present among the controls, with significant differences between the two conditions, as illustrated in Figure 2A,C.

PAC was substantially suppressing the proliferation at a rate of 60% in MDA-MB-231 and 30% in MCF-7 cells (Figure 2A,C). These results suggest that PAC is more effective in the triple-negative cell line.

In this study, the curcumin analog PAC was screened for its cytotoxic activities against BC cell lines MDA-MB-231 (TNBC) and MCF-7 (HR+ HER2-). LDH assay was performed to assess its cytotoxicity. It significantly caused an increase in LDH release in both after 24 h of treatment in comparison to the vehicle control. Our results shown in Figure 2D indicate that PAC exhibited potent cytotoxic activity for MDA-MB-231 (60%) and in the same manner, the outcomes were promising with MCF-7 (30%) as presented in Figure 2B,D. Consequently, PAC was more cytotoxic against MDA-MB-231 cells compared to estrogen receptor-positive MCF-7 cells.

### 2.3. PAC Suppresses the Migration in Breast-Cancer Cell Lines

**Figure 3:**
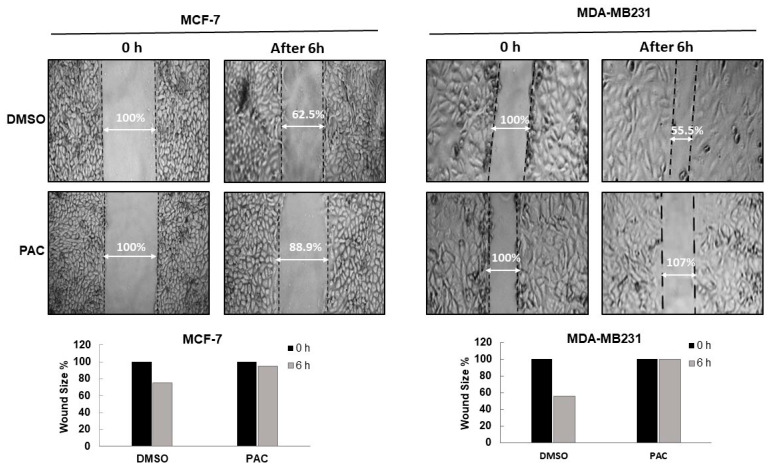
Effects of PAC on cell migration. Migration of MCF-7 and MDA-MB231 cancer cell lines was assayed by the wound-healing assay.

An increase in the migratory ability is one thing about a tumor cell that plays a critical role in its metastasis. Therefore, we examined the effect of PAC on motility in breast-cancer cell lines by a wound-healing assay for MCF-7 and MDA-MB-231. As shown in Figure 3, it is apparent that cellular migration was significantly inhibited by treatment with PAC in both cell lines. Status at 0 h versus 6 h demonstrated an increase in the migration of cells and a reduction over time in the wound size for the vehicle, reaching up to 44.5% in the MDA-MB-231 control while approximately 0% after 6-h exposure at 10 µM. In MCF-7 cells control, wound closure was 37.5% and 0% while MCF-7 was treated by 10 µM of PAC for 6 h. Our results show that PAC could have the ability to repress the motility of breast-cancer cells.

### 2.4. PAC Induces Greater Cell Apoptosis in Triple-Negative Breast Cancer

**Figure 4:**
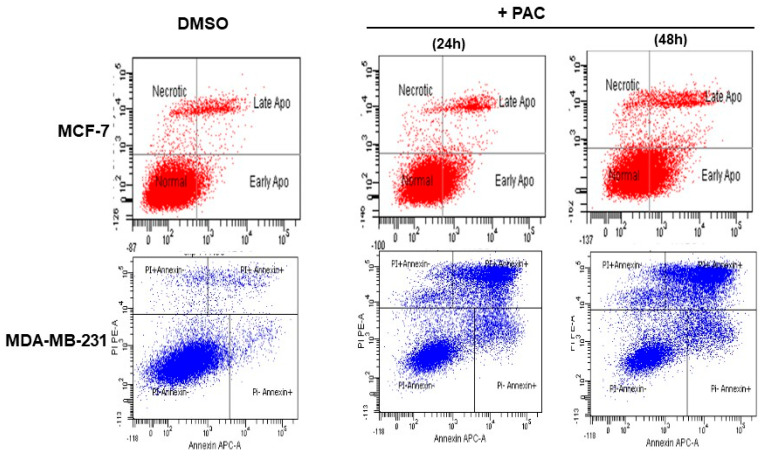
Effect of PAC on breast cancer cell apoptosis by flow-cytometry. The apoptosis rate of cells in each group was detected using flow cytometry by using Annexin/Pi assay for 24 h and 48 of PAC treatment.

To assess the apoptotic activity of PAC, the annexin V PI test was performed, and the cells were analyzed by flow cytometry. Figure 4 presents four different populations: live cells (annexin V-FITC−/PI−), early apoptotic cells (annexin V-FITC+/PI−), late apoptotic cells (annexin V-FITC+/PI+), and necrotic cells (annexin V-FITC−/PI+). Thus, it illustrates the percentage of cell death for MCF-7 and MDA-MB-231 after treatment with 10 µM of PAC. The apoptosis proportion was considered as the sum of the early and late stages. Around 20% of MCF-7 cells underwent apoptosis in response to 24 h of exposure and about 41% after 48 h with 10 µM of PAC while this same concentration on MDA-MB-231 cells for 24 h caused programmed cell death in 58.6%, and 73.2% after 48 h. It can be seen from the data in Figure 4 that apoptotic activity increased in a time-dependent manner. Importantly, these results indicate that PAC not only suppresses cancer cell proliferation but also triggers apoptosis mostly in triple-negative breast-cancer cells.

### 2.5. PAC Induces Apoptosis in MCF-7 and MDA-MB-231 through an Increase in the Bax/Bcl-2 Ratio

**Figure 5:**
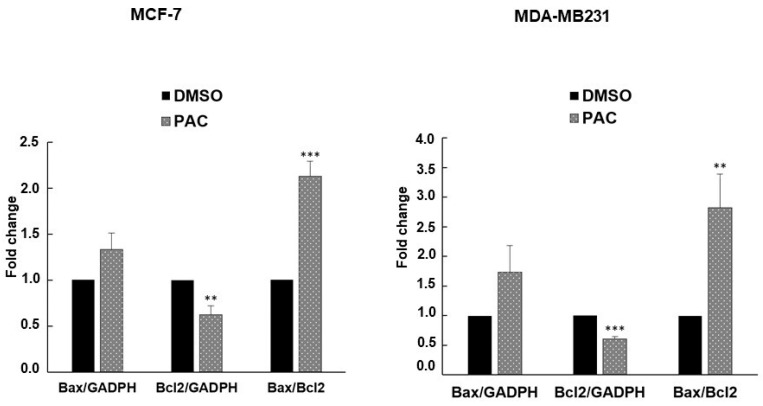
mRNA expression of Bax/Bcl-2. MCF-7 and MDA-MB231 Quantitative real-time PCR was performed to determine the expression level of Bcl-2 and Bax genes. GAPDH was used as a housekeeping gene. The results are expressed as mean ± S.D. (n = 4). ** *p* < 0.005 and *** *p* < 0.0005 versus control.

To validate the apoptotic status and possible molecular mechanism of PAC-induced cell death, the apoptosis-related genes (Bax and Bcl-2) were quantified at the mRNA level using RT-PCR analysis. Their degree of expression was assessed in MDA-MB-231 as well as in MCF-7 compared to controls (DMSO). The gene expression was normalized to GADPH. Our results suggest that the expression of the proapoptotic gene (Bax) in both cell lines was increased after treatment with 10 µM of PAC. Versus the vehicle control, our study also demonstrates that the antiapoptotic gene (Bcl-2) had a highly significant decrease after being treated with PAC. Consequently, PAC elevated the Bax/Bcl-2 ratio three times in MDA-MB-231 and twice in MCF-7, as shown in Figure 5.

Overall, these findings indicate that PAC promoted apoptosis through the upregulation of proapoptotic genes and the downregulation of antiapoptotic genes.

### 2.6. PAC Modulates the DNA Repair Gene Expression in MCF-7 and MDA-MB-231 Cells

**Figure 6:**
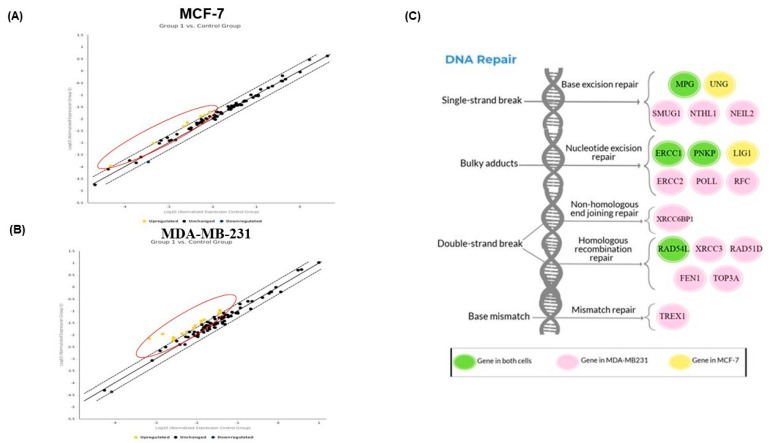
Screening using the RT^2^ Profiler PCR Array to determine the effect of PAC on genes involved in the DNA repair pathway. Scatter plots show the upregulation (yellow dots) by more than 2 fold compared with untreated control cells. (**A**) In MCF-7, six genes were upregulated. (**B**) In MDA, 16 genes were upregulated. (**C**) The schematic diagram represents the upregulation of DNA repair genes in MCF-7 and MDA-MB231cells because of treating them with PAC.

The expression of genes involved in DNA repair pathways was evaluated in MCF-7 and MDA-MB-231 by RT^2^ Profiler PCR Array (Figure 6). When MDA-MB-231 cells were undergoing treatment with PAC, our data show that a total of 16 genes were upregulated on 84 genes examined, with at least a two-fold change in gene expression between controls and treated cells (Figure 6B). Particularly, we further characterize these genes from different pathways: nucleotide excision repair (ERCC1, ERCC2, PNKP, RFC1, and POLL), base excision repair (MPG, NEIL2, NTHL1, and SMUG1), double-strand break repair (RAD51D, RAD54L, TOP3A, XRCC3, XRCC6BP1, and FEN1) and mismatch repair (TREX1). In addition, PAC led to a nine-fold increase in the expression level of XRCC6BP1 compared to the basal level. Similarly, that of XRCC3 was remarkably enhanced, about seven-fold higher than the baseline. In addition, this screening also indicated that PAC was able to upregulate the expression of six genes in MCF-7 cells treated in comparison to DMSO (Figure 6A). In the same manner, in MDA-MB-231, the upregulated genes in MCF-7 were classified as follows: nucleotide excision repair (ERCC1, LIG1, and PNKP), base excision repair (UNG and MPG) and double-strand break repair (RAD54L). Comparing the two sets of results reveals that the expression of the DNA repair genes, ERCC1, PNKP, MPG, and RAD54L was found to increase in both cell lines (Figure 6C).

### 2.7. Validation of DNA Repair in Gene Expression by RT-PCR Analysis

Gene-expression analysis of DNA repair pathways was further investigated to validate our previous findings. DNA repair genes were valued at the mRNA level using RT-PCR analysis. Similar results were obtained from the PCR array. Table 1 compares the data collected from preliminary analysis and the evidence from RT-PCR.

**Figure 7:**
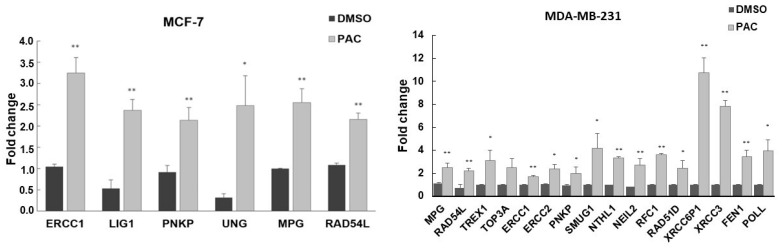
mRNA expression of DNA repair genes in MCF-7 and MDA-MB231. Quantitative real-time PCR was performed to determine the expression level of DNA repair genes. GAPDH was used as a housekeeping gene. The results are expressed as mean ± S.D. (n = 3). *, *p* < 0.05; **, *p* < 0.01 versus control.

As can be seen from Figure 7, when MDA-MB-231 cells were treated with PAC, this showed that genes involved in the nucleotide excision repair pathway (ERCC1, ERCC2, PNKP, POLL, and RFC1) were upregulated 1.69, 2.39, 2.00, 3.89, and 3.63 fold, respectively, while the expression of those from the base excision repair pathway (MPG, NEIL2, NTHL1, and SMUG1) increased 2.46, 2.74, 3.34, and 4.18 fold, respectively. As does the expression of the DNA repair genes that participate in the double-strand break repair pathway, it was found to be higher after treatment with PAC. RAD51D, RAD54L, TOP3A, XRCC3, XRCC6BP1, and FEN1 gene expression were remarkably enhanced, reaching 2.44, 2.21, 4.04, 7.82, 10.78, and 3.45-fold, respectively. In addition, PAC led to a 2.91-fold increase in the level of TREX1, a gene involved in the mismatch repair pathway. In MCF-7, PAC induced ERCC1, LIG1, PNKP, UNG, MPG, and RAD54L, approximately a two-fold increase in gene expression relative to the baseline, and the difference was statistically significant. These results indicate that PAC alters gene expression in the DNA repair pathway (Figure 7).

### 2.8. Interaction Network of All Associated Genes in Human DNA Repair

**Figure 8:**
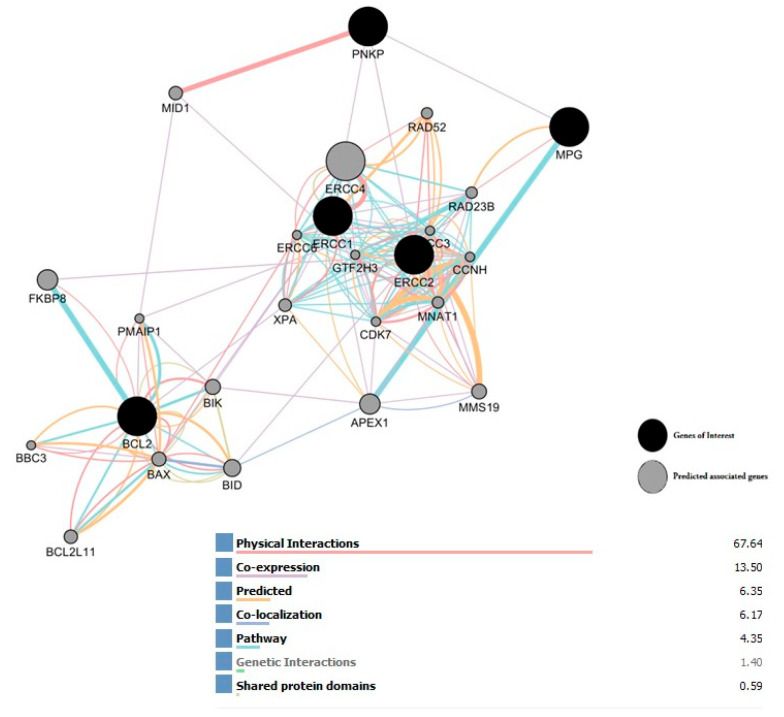
Interaction network of all associated genes in human DNA damage. An interaction network of all associated genes was used for analyzing gene–gene interactions. Input genes predict genes with their categorized functional association implied by genomic was determined by GENEMANIA (University of Toronto, Toronto, ON, Canada), was applied to conduct a network analysis of the common genes and to predict related genes and can be installed from ‘App Manager’ in Cytoscape software (Institute of Systems Biology, Seattle, WA, USA).

The Gene MANIA database was used to identify a gene interaction network. In addition, there were five genes (MPG, PNKP, ERCC1, ERCC2, and BCL2) to investigate all interactions between these genes. We selected five query genes (black circles); genes from the PCR array, they correspond to the genes of interest, along the interaction network of all that are connected. This network analysis of the common genes showed direct and indirect interactions among them via coexpression, genetic interactions, pathways, predicted and physical interactions, and shared protein domains with predicted associated genes (gray circles) indicating those that are more likely to be functionally related. Presented in Figure 8.

## 3. Discussion

In the current study, we examined the anticancer effects of PAC, a new curcumin analog, on breast cancer cells, especially those in the DNA repair system. It was reported that DNA damage causes cancer development, when erroneous repair is preventing it, particularly the mutations, and by activating oncogenes or inactivating tumor suppressor genes [16].

Firstly, we have found that PAC demonstrated stronger anticancer properties with a more potent effect on ER-negative cells by triggering apoptosis. In addition, the proapoptotic effect of PAC was sixfold higher against ER − (MDA-MB-231) than against ER + (MCF-7). This induction of apoptosis is mediated in the two breast-cancer cell lines through the mitochondrial pathway via the downregulation of Bcl-2/Bax. This outcome comes after that of Al-Hujaily et al. (2011) [11]. Thereby, PAC exhibited enhanced antimetastasis activity for both cell types, which gives this molecule the property of being a powerful adjuvant therapy for ER-negative breast cancers that are clinically aggressive. It is worth noting that many in vitro and in vivo studies reported that several synthetic analogs of curcumin are effective, such as EF24 having a strong anticarcinogenic effect against breast-cancer cells [17,18]. EF24 exhibits its anticancer activity by triggering cell-cycle arrest and apoptosis [19,20]. The proapoptotic effect of PAC is twofold higher than that of EF24 against MDA-MB-231 cells [19,20]. In addition, PAC showed a substantial immunomodulatory effect being more efficient against ER-negative than ER-positive cells. It was documented that PAC induces cancer-cell apoptosis more than EF24 [11]. Our previous study reported that it also does for toxicity in oral-cancer cells through the inhibition of NF-κB, MAPkase, and Wnt pathways, and by inducing oxidative stress [21].

Normal cells are characterized by their ability to preserve genome stability and integrity through capabilities to correct the impaired DNA that can contribute to carcinogenesis [22,23]. Numerous studies have widely documented the fact that the deregulation of the DNA repair mechanism is often linked to the initiation and progression of many cancers as well as to hypersensitivity/resistance of their cells to genotoxic agents [24]. Therefore, targeting the DNA repair pathways may be a powerful approach leading to an increase in the sensitivity of cancer cells to therapies. Our results show that PAC upregulated as a minimum a two-fold difference in gene expression between controls and treated cells, of which 6 DNA repair genes in MCF-7 and 16 in MDA-MB-231. These genes belong to nuclear and mitochondrial DNA repair pathways that include base excision repair (BER), nucleotide excision repair (NER), mismatch repair (MMR), and recombinational repair (homologous recombination and nonhomologous end joining) [25,26]. PAC induces six genes from the NER pathway. N-methylpurine DNA glycosylase (MPG) is shown to be overexpressed in both cell lines; however, the uracil DNA glycosylase (UNG) was upregulated only in MCF-7 while three other repair genes, single-strand selective monofunctional uracil-DNA glycosylase 1 (SMUG1), nth-like DNA glycosylase 1 (NTHL1), and nei-like DNA glycosylase 2 (NEIL2), were upregulated by treatment with PAC in MDA-MB-231. It has been noted that an elevated expression of MPG in certain types of cancer cells confers higher sensitivity to alkylation agents because MPG-induced apurinic/apyrimidinic (AP) sites trigger more strand breaks. This gene is known to bind directly to p53 (tumor-suppressor gene) to inactivate p53 in unstressed cells. In addition, the overexpression of MPG increases considerably, and the expression levels of the proarrest gene downstream of p53, including p21, but not proapoptotic ones. In addition, an overexpressed MPG gene has been reported to sensitize human breast-cancer cells [27] and ovarian-cancer cells [28] to a chemotherapeutic agent. In this current study, we hypothesize that the repair of DNA damage induced by PAC in both breast-cancer cell lines by the BER pathway starts with the recognition and removal of the damaged bases by N-methylpurine DNA glycosylase.

SMUG1 is known to remove uracil and certain oxidized bases from DNA during base excision repair (BER). MUG1 may play a role in preserving genome stability. Kemmerich K et al. (2012) reported that mice carrying a loss of SMUG1 and UNG in combination with loss of mismatch repair (Msh2 −/−) had shortened life spans and increased tumor formation, which implies a role for SMUG1 in protecting cells from the genomic instability that induces cancer, perhaps through direct removal of hmdU moieties [29]. The second BER-pathway gene upregulated by PAC in MDA-MB-321 was NTHL1, which comprised DNA glycosylases. Many studies have shown an average of 30,000 base lesions per cell removed daily by the DNA glycosylases of the base excision repair machinery [30]. It has been reported that germline mutations in these enzymes are directly associated with many diseases, including cancer [31,32,33]. Recently, numerous findings highlighted the importance of detectable variation in NTHL1 and its consequences in colorectal cancer among many ethnic groups [32,34]. Altered NTHL1 function can provoke genomic instability and cell transformation. In the present study, another gene of the DNA glycosylase family involved in the BER pathway has been flagged as being induced by PAC, only in triple-negative breast cancer; it is named NEIL1. Human NEIL1 glycosylases are known to participate in DNA repair by removing oxidative products of cytosine and protecting cells from damage [35]. This glycosylase takes part in the first step of BER. Numerous studies have indicated that any alteration in NEIL1 expression can lead to the deregulation of cell death and carcinogenesis [36] and reported that, in diverse cancers, the abnormal expressions of NEIL2 were widely associated with the somatic mutation load [37]. Secondly, our findings show six genes from the NER pathway induced by PAC exposure in breast-cancer cells, only ERCC1 and PNKP were observed in both types of cell lines, and only LIG1 was seen to be increased by PAC in MCF-7, while ERCC2, RFC1, and POLL were upregulated by treatment with PAC treatment in MDA-MD-321.

In humans, NER is considered the most versatile DNA repair system able to fix a variety of large lesions, including ultraviolet light-induced DNA photoproducts [37]. The consequences of defective NER associated with xeroderma pigmentosum (XP) were documented. Reports have indicated that patients with XP were extra sensitive to sunlight and often develop skin cancers at an early age [38,39]. Genetic polymorphism studies on NER showed evidence between this pathway and ovarian-cancer progression [40]. This outcome is in concordance with our results suggesting that upregulation by PAC of these genes involved in the NER pathway may be important in breast-cancer therapy. In addition, excision repair cross-complementing group 1 (ERCC1) is largely reportable as a key player in this pathway and has been studied extensively in many diseases, including nonsmall-cell lung cancer [41,42]. Recent evidence investigates the association between ERCC1 mRNA expression levels and clinical findings after treatment with a combination of gemcitabine and cisplatin for patients with advanced-stage nonsmall-cell lung cancer [43]. ERCC2 is a component of the NER pathway and plays a crucial part in the prediction of response to cisplatin in the bladder [44] and urothelial cancer [45]. Yang and al. (2018) have reported that, in traditional Chinese medicine, curcumin can sensitize human colon cancer to radiation by altering the expression of DNA repair-related genes, such as LIG4 and PNKP [46]. Tsukada et al. (2021) pointed out the importance of PNKP recruitment to DNA damage sites for the repair and maintenance of genome stability [47]. Many studies have shown that loss or variant of LIG1 is involved in genomic instability and tumorigenesis [48]. Another NER pathway gene was seen to be induced by PAC only in triple-negative breast cancer, termed replication factor C subunit 1 (RFC1). This is known to have a role in telomere stability [49] but the one in cancer therapy is not very clearly investigated.

Thirdly, our results demonstrate that PAC induces only one gene involved in nonhomologous end-joining repair; it is named XRCC6 binding protein 1 (XRCC6BP1) in triple-negative breast-cancer cells. Some studies point to the potential of targeting components of the DNA repair pathway, particularly XRCC6BP1, in chemo-resistant lung cancer. Furthermore, they concluded that it may play an important role in lung-cancer stem-cell subsets, at least in part; it may be responsible for chemotherapy resistance [50]. Five other members belonging to the family of homologous recombination repair genes were upregulated by PAC in triple-negative breast-cancer cells (RAD54L, XRCC3, RAD51D, FEN1, and TOP3A) but only RAD54L is common in both types of cell lines (MCF-7 and MDA-MB-231). Moreover, the RAD54L gene has been flagged as to be a candidate cosuppressor in breast or colon carcinomas, lymphomas, and meningiomas [51,52], supporting its role in tumorigenesis [53]. Andre et al. (2017) reported that RAD54L is altered in 0.74% of all cancers, such as colon, lung, and endometrial endometrioid adenocarcinoma, invasive ductal breast, and urothelial bladder carcinoma [54]. RAD54L is now considered a predictive biomarker for the use of olaparib in patients, particularly in prostate carcinoma. A recent work by Roos et al. (2018) revealed that the XRCC3 gene contributes to resistance to the chemotherapeutic drug temozolomide in glioblastoma cells. Using an isogenic glioma cell line, in which XRCC3 was downregulated by interference RNA, they demonstrated its importance as a possible target for therapeutic intervention [55]. Other studies have reported the association between single-nucleotide polymorphisms in the XRCC3 gene in hepatocellular carcinoma [56]. In the current research, we proved that PAC induces RAD51D only in MDA-MB-231 cells. This result agrees with the literature and with clinicians’ claims who advise the tested patients of having a genetic mutation or a variant in the RAD51D gene, which is likely pathogenic and was associated with an increased chance to develop numerous diseases, including breast and ovarian cancers [57]. Additionally, a structure specific for flap endonuclease 1 (FEN1) was observed to be induced by PAC in triple-negative breast-cancer cells. It was documented that dysregulation of expression or mutation in FEN1 in normal cells leads to provoking various diseases, such as cancers [58]. Moreover, our data were conflicting with the literature supporting its overexpression that contributes to drug resistance and it suggests targeting this gene, which has been verified as an effective strategy in mono- or combination therapy for many cancers [59]. Our information is in contradiction with previous studies considering FEN1 as a marker of multiple metastatic tumors and poor prognosis [60] and is viewed as an effective target for tumor resistance treatment [61]. They propose that the development of efficacious small-molecule inhibitors targeting FEN1 would be an excellent strategy to treat various cancers [59,62]. Concerning a gene and its association with cancer progression, it was observed that the TOP3A mutation is a prognostic factor for adjuvant chemotherapy in triple-negative breast cancer [63]. Bai et al. (2016) strongly supported that TOP3A was expected to be further exploited as a tumor suppressor and is of crucial importance for the diagnosis, treatment, and prognosis of patients with epithelial ovarian carcinoma [64].

Finally, our results show that one gene in the mismatch repair pathway (three prime repair exonuclease 1 = TREX1) was induced by PAC in triple-negative breast-cancer cells. Lots of studies have found that, in cancer therapy, the dose-dependent efficacy of radiotherapy is at least partially attributable to TREX1 activity [65]. Our bioinformatic analysis indicates that these genes are connected by an interaction network. This network analysis of the common genes showed direct and indirect interactions among them via coexpression, genetic interactions, pathways, predicted, and physical interactions, and shared protein domains. This highly reliable network of functional interactions from DNA repair pathways can be used as the basic network or pathway-based analysis platform for cancer and other diseases.

## 4. Materials and Methods

### 4.1. Reagents and Chemicals

The MCF-7 and MDA-MB-231 human breast-cancer cell lines were obtained from ATCC Company (Manassas, VA, USA). DMEM (Dulbecco’s modified Eagle’s medium), FBS (fetal bovine serum), and antibiotics, P/S (penicillin/streptomycin), were from Thermo Fisher Scientific (Burlington, ON, Canada). As well, Lab-Tek chamber slides were purchased from Nalge Nunc, Inc. (Naperville, IL, USA). MTT solution, DMSO (dimethyl sulfoxide), trypan blue, and Triton X-100 were received from Sigma-Aldrich (Oakville, ON, Canada). PAC was provided by Dr. Basem Al-Otaibi and Dr. Ibrahim Al-Jammaz from King Faisal Specialist Hospital and Research Centre, Riyadh, Saudi Arabia. It was diluted in DMSO to reach a 10 mM stock solution and used at c. LDH-Cytotoxicity Colorimetric Assay Kit II was also needed (BioVision, Inc., Milpitas, CA, USA). Annexin V-FITC/Propidium Iodide Kit was from BD Biosciences (Mississauga, ON, Canada). RNA Extraction Kit and RT^2^ Profiler PCR Array Human DNA Repair (PAHS-042Z) were from Qiagen (Germantown, MD, USA). The High-Capacity cDNA Reverse Transcription Kit was from Applied Biosystems (Warrington, UK), and the SYBR Green Master Mix was from Bio-Rad Laboratories, Inc. (Hercules, CA, USA).

### 4.2. Cell Line and Culture

The breast-cancer cells were grown in DMEM with the addition of 10% FBS and 1% P/S, in 75 cm^2^ cell culture flasks. These were kept in a humidified atmosphere containing 5% CO_2_ and 95% air at 37 °C. The medium was changed every two days until 90% confluent.

### 4.3. Morphological Observation by Light and Fluorescence Microscopy

MCF-7 and MDA-MB-231 cells were seeded in 2-well Lab-Tek chamber slides with DMEM growth medium. After 24 h adhesion, the cells in one of the wells were treated with 10 μM PAC. Control cells in the second chamber were incubated with the same volume of DMSO-containing medium. After being treated for 24 h, the slides were observed under a phase-contrast microscope and photographed.

### 4.4. Cell Viability by MTT Assay

The cells were placed in 6-well plates at the density of 3 × 10^5^ cells/well, in 1000 µL of medium containing 10% FBS. The plates were incubated for one day at 37 °C under 5% CO_2_ in a humid atmosphere. Then, the cells were treated with PAC (10 µM) or DMSO (vehicle) for 24 h in a growth medium freshly prepared. Following this, the latter was removed after 24-h exposure to the drugs, and a fresh medium supplemented with 5 mg/mL MTT solution was added to each well and put in an incubator for three hours. At the end of this culture period, the supernatant was discarded and 500 µL of 0.04 N HCl in isopropanol was in turn used. Finally, the reaction product was quantified by measuring the absorbance with an xMark microplate reader from Bio-Rad at 550 nM. The proliferation percentage was evaluated in comparison with cells treated by DMSO. This experiment was repeated eight times.

### 4.5. Cytotoxicity Assay

The cytotoxic effect of PAC was calorimetrically determined with the LDH-Cytotoxicity Colorimetric Assay Kit II. Briefly, cells were treated as described above for the MTT assay and 100 µL of the supernatant was transferred into a 96-well plate according to the manufacturer’s instructions. After that, 100 µL of the reaction mixture was added and the plate was incubated at room temperature in the dark for 30 min. The reaction was stopped by adding 10 µL of stop solution. Finally, LDH release was quantified by measuring absorbance at 450 nm, the reference wavelength was 650 nm using an xMark plate reader. This experiment was done eight times.

### 4.6. Cellular Migration Analysis

A wound-healing assay was used as described by our previous works [16,17] to evaluate the effect of PAC on cell migration. Cells were seeded in 6-well plates to be 100% confluent. Once they achieve full confluence, they were treated with PAC (10 µM) or DMSO, and the monolayer was slowly and gently scratched with a 10 µL pipette tip. The images were taken at 0 h and 6 h, and the plates were incubated at 37 °C for one day under 5% CO_2_ in the humidified atmosphere. The migrating cells were quantified with wound closure metrics, by manually tracing the gap area in captured images, and the migration rate was expressed as a percentage of wound contraction. The photographs were analyzed by using ImageJ software.

### 4.7. Annexin V-FITC Propidium Iodide Assay

MCF-7 and MDA-MB-321 cells treated or not with 10 μM PAC, they were afterward incubated for 24 h in a 5% CO_2_ concentration at 37 °C. After 24 h of treatment, the cells were trypsinized, washed twice with PBS, and then stained with Annexin V and propidium iodide, following the manufacturer’s instructions, and next, the apoptotic cells were analyzed with a flow cytometry instrument (BD Biosciences, Mississauga, ON, Canada), as described by our previous works [66,67]. The experiment was repeated three times.

### 4.8. RNA Extraction and cDNA Preparation

Total RNA was extracted by using Qiagen RNA Extraction Kit per the manufacturer’s recommendation. The purity, concentration, and quality of the isolated RNA were all measured with a NanoDrop 8000 from Thermo Fisher Scientific. Next, 1 µg to 2 µg of total RNA was converted into cDNAs using a High-Capacity cDNA Reverse Transcription Kit according to the manufacturer’s directions, as described by our previous works [67,68,69].

### 4.9. Quantitative RT-PCR

As set out by our previous study [67], the expression of proapoptotic, and antiapoptotic factors, and selected DNA repair genes were assessed by quantitative RT-PCR with the SYBR Green Master Mix from Bio-Rad Laboratories, Inc., as required by the manufacturers. PCR was performed in a reaction volume of 25 μL. The following sequences were used as primers (Table 2 and Table 3).

Briefly, 12.5 µL SYBR Green, 0.5 µL primer (forward + reverse), 7 µL distilled water, and 5 µL cDNA sample, have been placed in MicroAmp Fast Optical 96-Well Reaction Plates. The plates prepared thereby were run on the 7500 Real-Time PCR System (Applied Biosystems). The PCR samples were preheated at 50 °C for 2 min and at 95 °C for 5 min, followed by 40 cycles (denaturing at 95 °C for 15 s, annealing at 62 °C for 30 min and extension at 72 °C for 30 s). Each reaction has been done in triplicate. By the presence of a single melt peak, the specificity of each primer pair had been verified. The cycle threshold (CT) for each sample was calculated from values using the assisting software from Applied Biosystems. The relative number of mRNA transcripts and the gene-expression quantification value were calculated after normalizing to that of the GAPDH housekeeping gene compared to control samples. Lastly, data were analyzed using the comparative cycle threshold method (2^−ΔΔCT^). The experiment was repeated four times.

### 4.10. Screening with RT^2^ Profiler PCR Array

To determine whether PAC modulates DNA repair pathways, real-time PCR was performed using RT^2^ Profiler PCR Array Human DNA Repair (PAHS-042Z) from Qiagen according to manufacturer instructions [70,71]. In addition, the PCR Array is a 96-well plate containing primers for a set of 84 DNA repair-related genes, five housekeeping genes, and three controls for the various ones included that have been investigated. This high-throughput approach for profiling has many features such as great sensitivity and specificity, a single gene-specific product in each reaction, and being highly reproducible. Moreover, it provides more meaningful data with higher precision and less time. Consequently, the RT^2^ Profiler PCR Array is considered the ideal tool for this study. Following the manufacturer’s instructions, after cDNAs were synthesized as described before, for each condition, the components of the PCR mixture were 1350 µL SYBR Green Master Mix, 102 µL cDNA, and 1248 µL RNase-free water; all were put in a 5 mL tube. Next, 25 µL of the PCR mix were added to each well of the RT^2^ Profiler PCR Array. The plate was centrifuged for 1 min at 1000× *g* at 25 °C. Then, the real-time PCR was performed. Lastly, relative gene expression was calculated using the 2^−ΔΔCT^ method. The experiment was repeated three times.

### 4.11. In Silico Analysis

A network of all associated genes was used in the study for analyzing gene–gene interactions. To make predictions with their categorized functional protein association implied by genomics, it was determined through GeneMANIA (University of Toronto, Toronto, ON, Canada), applied to conduct a network analysis of the common genes and to predict the related ones, it can be installed from the “App Manager” in the Cytoscape software (Institute for Systems Biology, Seattle, WA, USA). This network uses the GeneMANIA algorithm and combined data into five groups as follows: coexpression, genetic interactions, pathways, and predicted and physical interactions. GeneMANIA revealed this study that has found an interconnected network of five genes, either through physical interactions, coexpression, prediction, colocalization, pathway, genetic interactions, or shared protein domains that were subjected to gene–gene interaction network analysis using default parameters.

### 4.12. Statistical Analysis

All experiments were independently repeated at least three times and in triplicate. Data were analyzed with the Statistical Package for the Social Sciences (SPSS) for Windows. They are presented as the mean ± standard deviation (±SD) for continuous variables. For gene expression, the analysis was performed by using the comparative cycle threshold method (2^−ΔΔCT^), and the results were visually represented by fold changes.

## 5. Conclusions

Our data indicate that PAC upregulates multiple genes involved in numerous DNA repair pathways, which certainly can open a new horizon in triple-negative breast-cancer treatment. This compound can be used alone or with other drugs to make breast cancer cells more sensitive to radiation therapy/chemotherapy, and, above all, to reverse the resistance that 80% of these cells develop. Despite this potential, there are currently very few clinical trials testing the many curcumin analogs in combination with chemotherapy or radiotherapy.

## Figures and Tables

**Table 1:** The list of up-regulated genes in DNA repair signaling pathway in response to PAC treatment. (Fold change > 2, indicated gene up-regulation).

Cell Line	Genes	Name	Fold Regulation by RT^2^ Profiler PCR Array	Fold Regulation by RT-PCR
**MDA-MB 231**	ERCC1	Excision repair 1,endonuclease non-catalytic subunit	2.79	1.69
	ERCC2	Excision repair 1,TFIIH core complex helicase subunit	2.19	2.39
	PNKP	Polynucleotide kinase 3′-phosphatase	2.86	2.00
	POLL	DNA polymerase lambda	2.44	3.86
	MPG	N-methylpurine DNA glycosylase	2.54	2.46
	NEIL2	Nei like DNA glycosylase 2	2.28	2.74
	NTHL1	Nth like DNA glycosylase 1	2.81	3.34
	SMUG1	Single strand selective monofunctional uracil-DNA glycosylase 1	2.24	4.18
	RAD51D	RAD51 paralog D	2.23	2.44
	RAD54L	RAD54 like	2.43	2.21
	RFC1	Replication factor C subunit 1	2.23	3.63
	TOP3A	DNA topoisomerase III alpha	2.69	4.04
	XRCC3	X-ray repair cross complementing 3	7.32	7.82
	XRCC6BP1	XRCC6 binding protein 1	9.98	10.78
	FEN1	lap structure-specific endonuclease 1	2.57	3.45
	TREX1	Three prime repair exonucleases 1	2.23	2.91
**MCF-7**	ERCC1	excision repair 1, endonuclease non-catalytic subunit	2.53	2.71
	LIG1	DNA ligase 1	2.08	2.43
	PNKP	polynucleotide kinase 3′-phosphatase	2.30	2.13
	UNG	uracil DNA glycosylase	2.12	2.48
	MPG	N-methylpurine DNA glycosylase	2.15	2.22
	RAD54L	RAD54 like	2.18	2.15

**Table 2:** Primer sequences used for gene expression of an internal control gene (GADPH) and proapoptosis and antiapoptosis (Bax, Bcl-2).

Gene	Strand	Primers Sequences	Amplicon Size (bp)	Tm
**GAPDH**	Forward	GGTATCGTGGAAGGACTCATGAC	188	66
	Reverse	ATGCCAGTGAGCTTCCCGTTCAGC		
**Bax**	Forward	AGTGTCTCAGGCGAATTGGC	102	60
	Reverse	CACGGAAGAAGACCTCTCGG		
**BcL2**	Forward	ACTGAGTACCTGAACCGGCATC	108	61
	Reverse	GGAGAAATCAAACAGAGGTCGC		

**Table 3:** Primer sequences were used for the validation of selected DNA repair genes in this study.

Gene	Strand	Primers Sequences	Amplicon Size (bp)	Tm
**ERCC1**	Forward	GGGTGACTGAATGTCTGACCA	191	54
	Reverse	GGGTACTTTCAAGAAGGGCTC		
**ERCC2**	Forward	GACCTGGTGTCCAAGGAACTG	165	57
	Reverse	GATCCTGAGCACCGTCTTCTG		
**FEN1**	Forward	AAGTCTATGCTGCGGCTACC	83	60
	Reverse	TTCACTGGCAGTCAGGTGTC		
**MPG**	Forward	GAAGCAGCG ACCAGCTAGAGC	83	60
	Reverse	CGAGCTGTGTGGCTGCTCT		
**NTHL1**	Forward	CAAGATGGCACACCTGGCTA	174	60
	Reverse	TCGTGCCACAGCTCCCTA		
**PNKP**	Forward	GTTACGGACCGGAAGTGCTCCA	120	60
	Reverse	CTTCAACTCCTGGGTCCCGGTA		
**POLL**	Forward	CATTTGCCCTCCTTCCCCC	144	60
	Reverse	GCCCCAAGAGTCTCTCCAAC		
**RAD51D**	Forward	AGTGGTGGACCTGGTTTCTG	139	60
	Reverse	CTCGTAGAGATCAGCGCCATT		
**RAD54L**	Forward	TTCGATTGACCCGGTCTTGG	197	60
	Reverse	TCAGACTCAGGGAGGTCGAG		
**SMUG1**	Forward	AAGGAACCGGAAACGGGATG	109	59
	Reverse	GCAGCAGTCTCTTGATGAGG		
**NEIL2**	Forward	CCCTTCCGCGTCTCATCTTT	336	60.5
	Reverse	TTCCATGGACCTGGGTGTCC		
**TREX1**	Forward	GAGGGAGACCCTCTCAGACA	146	60
	Reverse	TGGGCAGAAGCACATCTCAG		
**RFC1**	Forward	ACAAACTCCTACGCTGGCTC	76	59
	Reverse	AATTTACCAAACTTTGCGTGTTTTT		
**TOP3A**	Forward	ATGAGGCGGAGAGAAGGACT	132	60
	Reverse	CTGCATCTGGAAATCATGAGCC		
**XRCC6P1**	Forward	CTCCAGCTTCTTCACCAGCA	113	59.5
	Reverse	CAGCACAACCTGAGTGTTTCA		
**XRCC3**	Forward	CGGGAGGATGTGCACAGAG	171	60
	Reverse	CTGTCACTCTCTGGGGCTTG		
**UNG**	Forward	CTCTGTGCAGGGTTCCCAGTC	345	63
	Reverse	GCTCACAACCAGACAGCTCC		
**LIG1**	Forward	AGGAGGCATCCAATAGCAGCA	87	61
	Reverse	GGACTTCCTGAATGGTCCGT		

## Data Availability

Not applicable.
